# Formal and informal mental health support in young adults with recurrently depressed parents

**DOI:** 10.1192/bjo.2025.10819

**Published:** 2025-09-09

**Authors:** Rhys Bevan-Jones, Bryony Weavers, Tessa Lomax, Emma Meilak, Olga Eyre, Victoria Powell, Becky Mars, Frances Rice

**Affiliations:** Wolfson Centre for Young People’s Mental Health and Division of Psychological Medicine and Clinical Neurosciences, Cardiff University, Cardiff, UK; Cwm Taf Morgannwg University Health Board, Mountain Ash, UK; Oxford Health NHS Foundation Trust, Warneford Hospital, Oxford, UK; Department of Psychiatry, University of Oxford, Warneford Hospital, Oxford, UK; Centre of Academic Mental Health, Population Health Sciences, University of Bristol, Bristol, UK; NIHR Bristol Biomedical Research Centre, University Hospitals Bristol and Weston NHS Foundation Trust and University of Bristol, Bristol, UK

**Keywords:** Mental health, support, service use, young adults, parental depression

## Abstract

**Background:**

A family history of mental illness, particularly parental depression, is a risk factor for mental health difficulties in young people, with this heightened risk extending into adulthood. Evidence suggests low rates of formal mental health support in children/adolescents with depressed parents, but it is unknown whether this pattern persists into adulthood and applies to informal support.

**Aims:**

We examined the prevalence of formal and informal mental health support accessed by young adults with recurrently depressed parents. We identified factors associated with access to different support, and report satisfaction with support.

**Method:**

The sample included 144 young adults (mean age 23 years, range 18–28 years) who completed psychiatric assessments and reported on their use of mental health support in a cross-sectional analysis of a longitudinal cohort study (wave 4). Regression analyses explored predictors for support.

**Results:**

Young adults accessed a range of formal (29%) and informal (56%) support. Among those with a psychiatric disorder, nearly half had not accessed formal support and a fifth had not accessed any support. Predictors of support included psychiatric disorder, severity indicators (e.g. self-harm/suicidal thoughts, impairment) and demographic factors (e.g. education, gender). Predictors varied by type of support. Most participants reported satisfaction with support.

**Conclusions:**

Young adults at high risk of mental disorders accessed various mental health support. However, many did not access/receive support when needed. Further work is required to improve access to tailored support.

Young people whose parents experience depression represent a recognised high-risk group for mental health difficulties,^1^ most commonly depression and anxiety,^2,3^ and the period of increased risk extends into early adult life.^4^ Children of parents who experience recurrent depression (i.e. at least two episodes of depression) are at a particularly high risk,^5,6^ and rates of recurrence in adults treated for depression are high.^7^ Previous research suggests low rates of use of formal support (e.g. health services) in this population as children and adolescents,^8^ but it is unknown whether this pattern continues into adult life. Indeed, young adult life is the peak period of onset for many mental health difficulties,^9,10^ including in the adult children of parents with recurrent depression.^4^ Moreover, there is increasing interest in young adult mental health in a clinical and research context, especially in ensuring access to support and in the development of appropriate services and resources.^11^ Studies to date have also focused primarily on formal health service use in this population. Given the pressure on formal services, there is greater appreciation of the importance of informal forms of mental health support, such as self-help, online approaches and social networks.^12–14^

It is well established that there is a significant treatment gap for mental health difficulties, including in young adults, influenced by demographic factors such as socioeconomic patterning and lower service use in young males.^14–19^ Predictors of access to support include the presence of comorbidity, self-harm/suicidal thoughts, severity of illness and impairment.^17–21^ Several potential facilitators and barriers to access have been identified, including individual, societal and service/support-related factors.^15,16,22,23^ A better understanding of the patterns of use of the range of services, resources and social networks, alongside the facilitators and barriers to accessing support, is important. This could inform strategies to improve access for young adults at risk of mental health difficulties, and help address the need identified in several international reports for the special consideration of early detection and intervention approaches for children of parents with mental illness, particularly depression.^1–3^

## Aims

This study focuses on a sample of young adults whose parents had been treated in primary care for depression. The aims were to:

- examine the prevalence of access to support for mental health difficulties in young adults;
- describe the types of support (e.g. services, resources, social networks) accessed;
- identify factors associated with use of support, and explore satisfaction with services.

## Method

### Participants

The sample includes young adults from the Early Prediction of Adolescent Depression (EPAD) study, a prospective longitudinal study of the children (born between 1990 and 1998) of parents with recurrent depression.^4,24–27^ The baseline sample included 337 parents (315 mothers, 22 fathers) and their biological children (aged 9–17 years, mean (s.d.) 12.4 (2.0) years) – 197 females and 140 males.

Parents and offspring were assessed separately via interview and completed questionnaires at four time points between April 2007 and September 2020. This paper focuses on the data from the fourth wave of collection, which occurred on average 10 years after baseline. Of the baseline sample, 73 families were not contactable at wave 4 due to either loss of up-to-date contact details, withdrawal from the study, death or declining to participate (due to ill health, bereavement or other commitments such as work), and 67 were then unresponsive despite multiple communication attempts.^4^ Of the 197 participants in wave 4, 144 young adults took part in an interview and provided data on support accessed. This included 89 females and 55 males, with an age range of 18–28 years (mean 23.5 years, s.d. 2.30 years). Most of the sample (*n* = 137, 95%) had two British parents and seven had either a mixed (*n* = 2) or unknown (*n* = 5) ethnic background.

The authors assert that all procedures contributing to this work comply with the ethical standards of the relevant national and institutional committees on human experimentation, and with the Helsinki Declaration of 1975 as revised in 2013. The study was approved by the Multi-Centre Research Ethics Committee for Wales (reference no. 06/MRE09/48) and the School of Medicine Ethics Committee, Cardiff University (reference no. 18/12). Written informed consent was obtained.

### Procedure

Participants were recruited primarily from general practices in south Wales (78% from 62 practices), with the remainder recruited from a database of individuals with previously identified unipolar depression (19%) and other sources (e.g. notice boards in primary care centres, 3%).^4,24–27^ At the time of recruitment, parents were screened over the telephone to ensure that they met the inclusion criteria: (a) a history of recurrent depression, defined as at least two episodes of depression (DSM-IV Major Depressive Disorder, MDD^28^), later confirmed at baseline using the diagnostic interview, Schedules for Clinical Assessment in Neuropsychiatry (SCAN);^29^ and (b) had a biologically related child aged 9–17 years and living at home. Families were excluded if the parent had a diagnosis of bipolar or psychotic disorder at baseline or if the child had a moderate to severe learning disability (IQ < 50). If there was more than one eligible child in the household, the youngest child was selected for participation. Most assessments took place in the participant’s home, with young adults and parents interviewed separately. A small number of assessments were undertaken via telephone/video call as required. Families were offered gift vouchers for participating in the study. More detail regarding recruitment, sample characteristics and assessments of the EPAD cohort can be found in other publications.^4,24–27^

### Measures

#### Mental health support

Participants were given a list of support sources and asked to indicate (yes/no) whether they were ‘currently seeing or using’ any of these for help with mental health issues. They could also provide a free-text response under ‘someone else’ if not listed. Data were categorised into the binary variables ‘formal support’ and ‘informal support’. Formal support included primary care (general practitioner), secondary care (mental health specialist: psychiatrist, clinical psychologist, mental health nurse) or other formal support (e.g. counsellor, social services, student support). Informal support included self-help (e.g. internet-based therapy/apps, self-help group), internet use (for information or advice) or family member or close friend.

Participants were asked whether they were satisfied with the help received if they had ‘ever used services for help with mental health’ (options: yes, no, not applicable) and why (free-text response). This was asked about help from services in general, and not for each type of support individually.

#### Predictors of support

Predictors were selected based on prior literature.^14–21^

Current psychiatric diagnoses: these were assessed using a semi-structured diagnostic interview, the Young Adult Psychiatric Assessment (YAPA).^30^ YAPA was used in separate interviews with parents and young adults to assess offspring DSM-IV^28^ psychopathology in the preceding 3 months. The parent interviews asked about symptoms of depression and attention-deficit hyperactivity disorder (ADHD) in their offspring, whereas the young adult interviews included assessment of a wide range of psychiatric disorders. Cases where the young adults met criteria for a psychiatric disorder or had subthreshold symptoms were reviewed by two psychiatrists, and diagnoses were agreed by clinical consensus.

Three variables were considered as predictors of service use: a diagnosis of any psychiatric disorder, a depressive disorder and an anxiety disorder, because these are the most common disorders in this population.^4^

A diagnosis of ‘any psychiatric disorder’ included depressive disorders (MDD, dysthymia, cyclothymia and adjustment disorder), anxiety disorder (generalised anxiety disorder (GAD), social anxiety, separation anxiety, agoraphobia, obsessive–compulsive disorder and panic disorder), ADHD, conduct disorder and personality disorders (schizotypal and borderline). Although personality disorders were not explicitly assessed by the standardised interview used, in a small number of cases a personality disorder diagnosis was judged by clinical consensus to be appropriate for the symptoms exhibited.

Comorbidity: this was defined as those currently meeting diagnostic criteria for two or more DSM-IV disorders, and categorised as a binary variable (yes/no).

Self-harm or suicidal thoughts: the presence of self-harm/suicidal thoughts over the past 3 months was assessed using YAPA. Responses to these questions were combined and categorised as a binary variable (yes/no).

Total difficulties and impairment: measures of total difficulties (total score, continuous) and impairment (impairment score, continuous), associated with emotional or behavioural problems, were indicated by the self-report Strengths and Difficulties Questionnaire (SDQ) impact supplement.^31^

Demographic factors: these included: (a) gender (female/male/other), (b) age in years, (c) poor social support (i.e. only one person or no one to rely on), (d) living alone, (e) not in education, employment or training (NEET), (f) education status (not completed degree and not currently in university) and (g) low personal income (categorised as below £18 000/annum^32^).

For further details on the measures, see Supplementary Material, Supplement 1 available at https://doi.org/10.1192/bjo.2025.10819.

### Analysis

We first describe the sample characteristics (proportions or means as appropriate) in the whole sample. Next, we describe the proportions of participants using different types of support in the whole sample and separately by psychiatric disorder status, because those meeting criteria for a disorder are more likely to require support.

A series of univariable logistic regression analyses were then conducted to investigate predictors of the three support outcome variables (any formal support, any informal support, any support (formal and informal combined)), first in the whole sample and then in the subsample with psychiatric disorder. Analyses reported in main text use inverse probability weighting (IPW)^33^ to account for attrition between study baseline and the fourth follow-up phase, the focus of this analysis. IPWs were calculated by examining variables at the baseline assessment that predicted missingness from the analysis sample consistent with previous publications^4^ (Supplement 2). The tables report results using IPW. Results were broadly similar when analysing complete cases and IPW (Supplement 3). A sensitivity analysis was conducted, excluding family/friends support from the informal support category, to examine sources developed to provide self-guided support, and because social support was included as a predictor. Quantitative data on satisfaction with services are presented descriptively (percentages), and free-text responses on reasons for satisfaction/dissatisfaction were categorised into broad themes. Statistical analysis was conducted using SPSS version 27 for Windows (IBM, Armonk, NY, USA; www.ibm.com/products/spss-statistics).

## Results

### Prevalence of mental health difficulties and demographic factors

Table 1 shows the psychiatric and demographic characteristics of the sample. Over a third (38.7%) of individuals in the sample met the criteria for a current psychiatric disorder, with 24.7% having a depressive disorder and 25.2% an anxiety disorder. Comorbidity was identified in 17.2% of individuals, and 12.4% had recent self-harm/suicidal thoughts. A quarter of the sample (24.7%) reported poor social support and 13.1% were living alone. Over 16% were NEET, and 43.1% had not completed a degree and were not currently in university. Over two-thirds (68.5%) had a personal annual income under £18 000.

**Table 1:** Prevalence of mental health difficulties and demographic factors (IPW applied)

Difficulties and factors	Full sample, % or mean (s.d.) *N* = 144
Mental health difficulties	
Any current psychiatric disorder	38.7
Any current depressive disorder	24.7
Any current anxiety disorder	25.2
Current comorbidity	17.2
Current self-harm or suicidal thoughts	12.4
SDQ total difficulties score	11.7 (5.99)
SDQ impairment score	1.5 (2.25)
Social, educational and occupational factors	
Gender (female)	61.8
Age (years)	23.5 (2.30)
Poor social support	24.7
Living alone	13.1
Not in education, employment, or training	16.1
Not completed degree and not currently in university	43.1
Personal income <£18 000 per annum	68.5

IPW, inverse probability weighting; *N*, number of young adults; SDQ, Strengths and Difficulties Questionnaire.

### Prevalence of access to support

Table 2 provides information about the use of different types of support. Among the whole sample, 60.2% of individuals reported currently receiving some form of mental health support. Informal support was used by a greater proportion than formal support (55.9 versus 29.3%). With regard to formal support, primary care was most frequently used (23.0%), followed by secondary care (10.3%) and other formal support (7.2%). For informal support, family/friends was the most reported (55.9%), followed by the internet (19.1%) and self-help (4.4%). When excluding family/friends support, the proportion accessing informal support reduced to 22.3%.

**Table 2:** Support accessed for mental health difficulties in the whole sample, and in those with and without a current psychiatric disorder (IPW applied)

Type of support accessed	Whole sample % *N* = 144	Any psychiatric disorder % *n* = 53	No psychiatric disorder % *n* = 91
Formal support			
Medical support			
Primary care	23.0	44.8	9.1
Secondary care	10.3	21.4	3.1
Medical support (total)	27.3	52.9	11.2
Other formal support	7.2	13.7	3.0
Any formal support (total)	29.3	56.5	12.1
Informal support			
Self-guided support			
Self-help	4.4	9.7	1.1
Internet	19.1	31.4	11.4
Self-guided support (total)	22.3	37.9	12.5
Family member or close friend	55.9	72.0	43.4
Any informal support (total)	55.9	74.3	44.3
Any support total (formal or informal)	60.2	82.0	46.4
Any support (excluding family members/friends)	39.3	68.7	20.7

IPW, inverse probability weighting; *N*, number of young adults; any formal support (total): primary care, secondary care or other formal support; any informal support (total): self-help, internet or family member/close friend; any support total: any formal or informal support.

Access to support was higher among those with a disorder compared with those without (formal, 56.5 versus 12.1%; informal, 74.3 versus 44.3%; any support, 82.0 versus 46.4%). However, 43.5% of those with a disorder were not in contact with formal services and 18.0% did not receive any support, or 31.3% when excluding family/friends support. Among those with a disorder, family/friends was the most reported type of support (72.0%) followed by primary care (44.8%) and the internet (31.4%). Again, the proportion of those with a disorder accessing informal support was lower (37.9%) when discounting family/friends support.

### Predictors of support in the whole sample

Findings from the regression analyses in the whole sample are shown in Table 3. Several variables were consistently associated with both formal and informal support. These included the presence of any psychiatric disorder, depressive disorder, anxiety disorder, self-harm/suicidal thoughts and higher SDQ difficulties and impairment scores. For education status, those without a degree and not in university were less likely to access formal support, and there was weak evidence for a negative association with informal support. There was weak evidence for an association with gender, with females slightly more likely to access both types of support than males. The odds ratios were often higher for formal than informal support, particularly for the disorder categories, although there was some overlap in the confidence intervals.

**Table 3:** Regression analysis on current support accessed by young adults in the whole sample (*N* = 144) (IPW applied)

	Any formal support		Any informal support		Any support	
Parameters	Odds ratio (95% CI)	*P*-value	Odds ratio (95% CI)	*P*-value	Odds ratio (95% CI)	*P*-value
Any current psychiatric disorder	9.4 (5.46–16.12)	<0.001	3.6 (2.25–5.85)	<0.001	5.3 (3.13–8.91)	<0.001
Any current depressive disorder	5.9 (3.46–10.09)	<0.001	2.2 (1.30–3.74)	0.003	4.1 (2.20–7.47)	<0.001
Any current anxiety disorder	6.4 (3.77–10.99)	<0.001	4.8 (2.65–8.74)	<0.001	5.5 (2.86–10.47)	<0.001
Current comorbidity	7.0 (3.77–12.85)	<0.001	1.5 (0.85–2.76)	0.15	3.6 (1.77–7.32)	<0.001
Current self-harm/suicidal thoughts	3.5 (1.80–6.78)	<0.001	3.19 (1.48–6.88)	0.003	2.6 (1.22–5.65)	0.014
SDQ total difficulties score	1.2 (1.12–1.23)	<0.001	1.1 (1.06–1.15)	<0.001	1.1 (1.09–1.20)	<0.001
SDQ impairment score	1.6 (1.39–1.84)	<0.001	1.4 (1.18–1.55)	<0.001	1.4 (1.21–1.64)	<0.001
Gender (female)	1.5 (0.94–2.51)	0.09	1.5 (0.96–2.31)	0.08	1.5 (0.93–2.25)	0.10
Age (years)	0.95 (0.85–1.05)	0.29	0.98 (0.89–1.07)	0.61	0.99 (0.90–1.09)	0.87
Poor social support	1.2 (0.69–2.11)	0.51	2.2 (1.33–3.66)	0.002	1.6 (0.95–2.59)	0.08
Living alone	2.7 (1.36–5.22)	0.004	0.8 (0.44–1.63)	0.61	2.1 (0.98–4.34)	0.06
Not in education, employment or training	3.6 (1.96–6.79)	<0.001	1.2 (0.65–2.23)	0.56	2.1 (1.05–4.05)	0.04
Not completed degree and not currently in university	0.4 (0.23–0.61)	<0.001	0.7 (0.43–1.03)	0.07	0.5 (0.31–0.76)	0.002
Personal income <£18 000 per annum	1.3 (0.66–2.41)	0.49	1.7 (0.97–2.90)	0.06	1.9 (1.08–3.24)	0.03

IPW, inverse probability weighting; SDQ, Strengths and Difficulties Questionnaire.

Comorbidity, living alone and NEET were associated only with formal support whereas poor social support was associated only with informal support. There was weak evidence for an association between low personal income and informal support. Age was the only variable that was not associated with either type of support. In sensitivity analysis (Supplement 4) excluding family/friends support from the informal support outcome, there were associations with comorbidity and NEET in addition to those noted above. However, associations were no longer found with self-harm/suicidal thoughts, gender and education status (previously weak evidence).

### Predictors of support in young adults with a psychiatric disorder

Findings from the regression analyses among those with a disorder are shown in Table 4. Those with comorbidity and greater SDQ total difficulties and higher impairment scores were more likely to access formal support. Those without a degree and not in university and those with a low personal income were less likely to access formal support.

**Table 4:** Regression analysis on current support accessed by young adults with a psychiatric disorder (*N* = 53) (IPW applied)

	Any formal support		Any informal support		Any support	
Parameters	Odds ratio (95% CI)	*P*-value	Odds ratio (95% CI)	*P*-value	Odds ratio (95% CI)	*P*-value
Current comorbidity	2.02 (0.99–4.12)	0.05	0.39 (0.18–0.88)	0.02	0.99 (0.40–2.42)	0.97
Current self-harm/suicidal thoughts	0.69 (0.39–1.86)	0.69	0.93 (0.40–2.32)	0.16	0.49 (0.19–1.26)	0.14
SDQ total difficulties score	1.15 (1.07–1.25)	<0.001	1.02 (0.95–1.10)	0.64	1.09 (1.00–1.19)	0.05
SDQ impairment score	1.41 (1.18–1.69)	<0.001	1.18 (0.99–1.41)	0.06	1.16 (0.96–1.40)	0.13
Gender (female)	0.89 (0.44–1.79)	0.73	1.80 (0.82–3.97)	0.15	1.26 (0.51–3.09)	0.62
Age (years)	0.94 (0.81–1.09)	0.40	0.87 (0.73–1.02)	0.09	0.85 (0.70–1.03)	0.10
Poor social support	1.88 (0.85–4.16)	0.12	4.24 (1.81–9.94)	<0.001	2.39 (0.93–6.11)	0.07
Living alone	2.42 (0.88–6.65)	0.09	0.44 (0.16–1.17)	0.10	2.89 (0.61–13.63)	0.18
Not in education, employment or training	1.48 (0.68–3.20)	0.33	0.20 (0.08–0.48)	<0.001	0.43 (0.17–1.07)	0.07
Not completed degree and not currently in university	0.34 (0.17–0.71)	0.004	0.62 (0.28–1.36)	0.23	0.16 (0.06–0.48)	0.001
Personal income <£18 000 per annum	0.32 (0.11–0.88)	0.03	2.61 (0.83-8.21)	0.10	2.61 (0.83–8.21)	0.10

IPW, inverse probability weighting; SDQ, Strengths and Difficulties Questionnaire.

Those with comorbidity and who were NEET were less likely to access informal support. There was weak evidence for an association with SDQ impairment and a negative association with age. Those with poor social support were more likely to access informal support, and this association remained (although weaker) after sensitivity analysis when excluding family/friends from informal support (Supplement 4). A similar pattern of sensitivity analysis results was found for SDQ impairment and age, although associations were no longer found for comorbidity or NEET. Additional associations were found – those with self-harm/suicidal thoughts and living alone were less likely to access support and those with higher SDQ difficulties scores were more likely.

### Satisfaction with services

Ninety-three young adults (64.6% of the whole sample) reported having ever used services for help with mental health, and provided information on satisfaction with those services. Of these, over two-thirds (69.6%) were satisfied with the help received and 21.5% were not satisfied. The remainder answered both ‘yes’ and ‘no’ (6.5%) or ‘don’t know’ (2.2%).

The most common reasons given for satisfaction with services (Supplement 5) included being taken seriously, feeling listened to and understood, being helped to rationalise, talking to someone impartial and speed of appointment. Reasons for dissatisfaction included long waiting times, disjointed services, feeling dismissed/unsupported, being offered medication too quickly and poor relationships with professionals.

## Discussion

This paper examined access to formal and informal types of mental health support in a sample of young adults at high risk of mental health difficulties due to a family history of recurrent depression. Sixty per cent across the whole sample reported access to some form of support and 29% used formal support. Although access to formal and informal support was higher among those with a psychiatric disorder, 44% of this group were not in contact with any formal services, and just under 1 in 5 received no support at all; this figure rises to about 1 in 3 when excluding social networks.

A wide range of formal and informal support were accessed. Access to support among the whole sample was predicted by diagnosis, as well as by other indicators of severity of difficulties (e.g. self-harm/suicidal thoughts, SDQ scores). Associations were consistently found for demographic predictors such as education status and gender. There were, however, some differences found for the remaining factors according to the type of support (formal/informal), and in the subsample with a disorder, which may include possible barriers and facilitators to accessing support. Over two-thirds were satisfied with the help received from services.

### Comparison with existing literature

Service use has previously been examined in this cohort when the participants were aged 9–17 years.^8^ At that time, although only a third of those with psychiatric disorder were in contact with services, only formal service use was examined, including educational, social, youth justice and health services. The current study builds on this work by including informal services and, with the focus on early adult life, a developmental transition to independence associated with the emergence of mental health difficulties,^9,10^ changes in support services and personal and social changes and challenges (e.g. education, employment, relationships).^34^ The current work also helps to address the lack of long-term studies in this population, and suggests that access to formal support among those with a disorder increases from childhood/adolescence to young adulthood (from a third to just over half the sample).

The levels of support accessed in this sample are higher than those reported in some earlier studies involving young adults with mental health difficulties in the UK. Salaheddin et al showed that 65% of 16- to 25-year-olds with mental health difficulties had accessed formal or informal help (including peer support).^15^ This compares with 82% of those with a disorder who accessed any support in our study, and might suggest that individuals with a parent (known to services) with mental health difficulties may be more likely to seek support, or could be explained by differences in methodology (e.g. participant characteristics, definition of difficulties and support).

Factors associated with access to support in the current study are consistent with those found in previous studies of young adults in the UK, such as mood disorders, severity of difficulties, comorbidity, suicide risk and female gender.^16,18,20^ The current study extends this work by looking at a wide range of sociodemographic factors (including age, social support, living alone, NEET, education status and personal income) and suggests that, for some of these factors, associations may be different for formal and informal support.

### Strengths and limitations

Data were drawn from a large study of young adults at elevated risk for psychopathology, recruited mainly from general practice, and who were followed prospectively over 13 years (from childhood/adolescence) and across key developmental phases. Assessments were rigorous, involving multiple informants and diagnostic interviews, and access to a broad range of support was considered.

The findings must, however, be interpreted considering the following limitations. The interview used to capture mental health support relied on the individual’s recall and interpretation of the question on whether they were currently receiving/using support. Therefore, some forms of support may have gone unreported. Broad definitions for support were used, although sensitivity analyses were conducted excluding social networks from informal support. Data on satisfaction with help received were based on lifetime reporting and were not specific to the type of services accessed. Social desirability bias may have led to over-reporting of satisfaction, although participants were not reporting back to service providers. A large number of analyses were conducted, and it is therefore possible that some results may have arisen by chance. Replication of these findings is needed in further studies.

As with all longitudinal studies, there was some attrition. Of the original 337 families in the sample, 197 (58.5%) took part in wave 4, with 144 participants having data on both disorder and support, leading to small subsamples for certain analyses (e.g. those with a disorder). However, IPW was used to account for attrition. Most of the parents in the cohort at baseline were depressed mothers (>90%), which may have affected the generalisability of the findings. This might reflect how research participation and help-seeking, in general, are lower in males.^17–21^ Further research is needed to understand risk of psychopathology and access to support in children of depressed fathers. The general strengths and limitations related to the EPAD cohort are also discussed in other publications.^4,24–27^

### Implications for practice

Young adults in this study accessed a range of support, and distinct levels and types of support are likely to be required based on individual needs and the severity of difficulties/impairment. UK guidelines for depression recommend tailored approaches including guided self-help, psychotherapy and medication, depending on the presentation from subthreshold/mild to severe depression.^35^ A UK study of older adolescents^36^ found beneficial treatment effects on depressive symptoms only in those who met the criteria for psychiatric disorder or who had high subthreshold symptoms and impairment, suggesting that this is a suitable threshold for formal services.

In this sample, 44% of young adults with a disorder were not accessing any formal support, suggesting that many in need of support are not receiving it. Access to support among this subgroup was predicted by indicators of severity and impairment, alongside demographic factors such as living alone. Specifically, those with lower education status and personal income were less likely to seek formal support, suggesting they represent a hard-to-reach group where targeted interventions could improve access. Education status was also negatively associated with formal support in the whole sample, while those who were NEET were more likely to access support. These findings may appear contradictory, but could be explained by higher levels of difficulties and impairment among those who are unemployed.

The proportion of participants with a disorder receiving support increased from 56.5% (formal support) to 82% when including informal support, highlighting the reliance on less formal sources. This can be explained in part by the lack of formal services and growth in self-help, online resources and digital devices, as well as young adults’ comfort with informal support, attributable to factors including accessibility, convenience, trust, confidentiality and stigma.^14,37^ This also reflects the importance of social networks, and the proportion accessing any support reduced to 68.7% following exclusion of family/friends.

Direct involvement of young adults is important to capture their lived experiences and inform both research and practice.^38^ To enrich our findings and guide future research in this area, a focus group was arranged with young adults to explore their experiences and perspectives on accessing support (Supplement 6). Of relevance to this study, they reported that their help-seeking behaviour could be influenced by how those close to them (e.g. parents/carers) managed their health difficulties and their experiences of accessing support. This aligns with previous findings suggesting that stigma associated with parental mental illness can deter family members from seeking support.^39^ The group also confirmed that young adults access a range of online and informal support and identified potential facilitators for help-seeking, including role models, public discussions about mental health and practitioner training. Reported barriers were consistent with those arising from the literature, including stigma,^15,17,22,23^ difficulty identifying or expressing concerns^15,21,23^ and uncertainty about where to seek help.^17^

The group’s recommendations for educational and anti-stigma initiatives reflect wider calls for help-seeking interventions to improve mental health knowledge and stigma.^37^ Participants stressed the need for improved coordination across health and student services and charities, and acceptable and effective resources and services that are co-developed with users.^40^ Further participatory work and mixed-methods research are required, particularly with children of depressed parents, to explore access to support.

In conclusion, all participants in this study were young adults with parents with recurrent depression and were therefore at elevated risk of mental health difficulties. Participants accessed a variety of sources of mental health support, with just over half of those with a psychiatric disorder accessing formal help, while one in five were not receiving any support. Further work is needed to ensure early identification of difficulties and access to support, and a better understanding of the types of support that meet the needs and preferences of young adults, including those at risk.

## Supporting information

Bevan-Jones et al. supplementary material Bevan-Jones et al. supplementary material

## Data Availability

Due to ethical restrictions, data collected at assessment waves 1–3 cannot be made openly available. Supporting data collected at assessment wave 4 are openly available from the Cardiff University data repository at http://doi.org/10.17035/d.2023.0263728184.

## Supplementary material

The supplementary material is available online at https://doi.org/10.1192/bjo.2025.10819

## References

[1] World Health Organization. Mental Health Action Plan 2013–2020. World Health Organization, 2013.

[2] Abel KM , Hope H , Swift E , Parisi R , Ashcroft DM , Kosidou K , et al. Prevalence of maternal mental illness among children and adolescents in the UK between 2005 and 2017: a national retrospective cohort analysis. Lancet Public Health 2019; 4: e291–300. 31155222 10.1016/S2468-2667(19)30059-3 PMC6557735

[3] Weissman MM. Children of depressed parents – a public health opportunity. JAMA Psychiatry 2016; 73: 197–8. 26841851 10.1001/jamapsychiatry.2015.2967

[4] Powell V , Lennon J , Bevan Jones R , Stephens A , Weavers B , Osborn D , et al. Following the children of depressed parents from childhood to adult life: a focus on mood and anxiety disorders. JCPP Adv 2023; 3: e12182. 38054049 10.1002/jcv2.12182 PMC10694536

[5] Brophy S , Todd C , Rahman MA , Kennedy N , Rice F. Timing of parental depression on risk of child depression and poor educational outcomes: a population based routine data cohort study from Born in Wales, UK. PLoS One 2021; 16: e0258966. 34788300 10.1371/journal.pone.0258966 PMC8598047

[6] Jaffee SR , Sligo JL , McAnally HM , Bolton AE , Baxter JM , Hancox RJ. Early-onset and recurrent depression in parents increases risk of intergenerational transmission to adolescent offspring. J Child Psychol Psychiatry 2021; 62: 979–88. 33222168 10.1111/jcpp.13356

[7] Ramanuj P , Ferenchick EK , Pincus HA. Depression in primary care: part 2—management. BMJ 2019; 365: l835. 30962249 10.1136/bmj.l835

[8] Potter R , Mars B , Eyre O , Legge S , Ford T , Sellers R , et al. Missed opportunities: mental disorder in children of parents with depression. Br J Gen Pract 2012; 62: e487–93. 22781997 10.3399/bjgp12X652355 PMC3381275

[9] Kessler RC , Angermeyer M , Anthony JC , De Graaf R , Demyttenaere K , Gasquet I , et al. Lifetime prevalence and age-of-onset distributions of mental disorders in the World Health Organization’s World Mental Health Survey Initiative. World Psychiatry 2007; 6: 168–76. 18188442 PMC2174588

[10] Solmi M , Radua J , Olivola M , Croce E , Soardo L , Salazar de Pablo G , et al. Age at onset of mental disorders worldwide: large-scale meta-analysis of 192 epidemiological studies. Mol Psychiatry 2022; 27: 281–95. 34079068 10.1038/s41380-021-01161-7 PMC8960395

[11] McGorry PD , Mei C , Dalal N , Alvarez-Jimenez M , Blakemore S-J , Browne V , et al. The Lancet Psychiatry Commission on youth mental health. Lancet Psychiatry 2024; 11: 731–74. 39147461 10.1016/S2215-0366(24)00163-9

[12] Goodwin J , Behan L , Kelly P , McCarthy K , Horgan A. Help-seeking behaviors and mental well-being of first year undergraduate university students. Psychiatry Res 2016; 246: 129–35. 27693865 10.1016/j.psychres.2016.09.015

[13] Pretorius C , Chambers D , Coyle D. Young people’s online help-seeking and mental health difficulties: systematic narrative review. J Med Internet Res 2019; 21: e13873. 31742562 10.2196/13873 PMC6891826

[14] Michelmore L , Hindley P. Help-seeking for suicidal thoughts and self-harm in young people: a systematic review. Suicide Life Threat Behav 2012; 42: 507–24. 22889130 10.1111/j.1943-278X.2012.00108.x

[15] Salaheddin K , Mason B. Identifying barriers to mental health help-seeking among young adults in the UK: a cross-sectional survey. Br J Gen Pract 2016; 66: e686. 27688518 10.3399/bjgp16X687313 PMC5033305

[16] Hodgson KJ , Shelton KH , van den Bree MBM. Mental health problems in young people with experiences of homelessness and the relationship with health service use: a follow-up study. Evid Based Ment Health 2014; 17: 76–80. 25043432 10.1136/eb-2014-101810 PMC13404263

[17] Ennis E , McLafferty M , Murray E , Lapsley C , Bjourson T , Armour C , et al. Readiness to change and barriers to treatment seeking in college students with a mental disorder. J Affect Disord 2019; 252: 428–34. 31003112 10.1016/j.jad.2019.04.062

[18] Duncan C , Rayment B , Kenrick J , Cooper M. Counselling for young people and young adults in the voluntary and community sector: an overview of the demographic profile of clients and outcomes. Psychol Psychother 2020; 93: 36–53. 30548244 10.1111/papt.12206 PMC7027817

[19] Lynch L , Long M , Moorhead A. Young men, help-seeking, and mental health services: exploring barriers and solutions. Am J Mens Health 2018; 12: 138–49. 27365212 10.1177/1557988315619469 PMC5734535

[20] Twomey CD , Baldwin DS , Hopfe M , Cieza A. A systematic review of the predictors of health service utilisation by adults with mental disorders in the UK. BMJ Open 2015; 5: e007575. 10.1136/bmjopen-2015-007575 PMC4499684 26150142

[21] Klineberg E , Biddle L , Donovan J , Gunnell D. Symptom recognition and help seeking for depression in young adults: a vignette study. Soc Psychiatry Psychiatr Epidemiol 2011; 46: 495–505. 20358174 10.1007/s00127-010-0214-2

[22] Pickles KJ , Rhind SM , Miller R , Jackson S , Allister R , Philp J , et al. Potential barriers to veterinary student access to counselling and other support systems: perceptions of staff and students at a UK veterinary school. Vet Rec 2012; 170: 124. 22186377 10.1136/vr.100179

[23] Cage E , Stock M , Sharpington A , Pitman E , Batchelor R. Barriers to accessing support for mental health issues at university. Stud High Educ 2020; 45: 1637–49.

[24] Mars B , Collishaw S , Smith D , Thapar A , Potter R , Sellers R , et al. Offspring of parents with recurrent depression: which features of parent depression index risk for offspring psychopathology? J Affect Disord 2012; 136: 44–53. 21962850 10.1016/j.jad.2011.09.002

[25] Mars B , Collishaw S , Hammerton G , Rice F , Harold GT , Smith D , et al. Longitudinal symptom course in adults with recurrent depression: impact on impairment and risk of psychopathology in offspring. J Affect Disord 2015; 182: 32–8. 25965693 10.1016/j.jad.2015.04.018

[26] Rice F , Sellers R , Hammerton G , Eyre O , Bevan-Jones R , Thapar AK , et al. Antecedents of new-onset major depressive disorder in children and adolescents at high familial risk. JAMA Psychiatry 2017; 74: 153–60. 27926743 10.1001/jamapsychiatry.2016.3140

[27] Powell V , Agha SS , Jones RB , Eyre O , Stephens A , Weavers B , et al. ADHD in adults with recurrent depression. J Affect Disord 2021; 295: 1153–60. 34706428 10.1016/j.jad.2021.09.010 PMC8552915

[28] American Psychiatric Association. Diagnostic and Statistical Manual of Mental Disorders (DSM-IV) (4th edn). APA, 1994.

[29] Wing JK , Babor T , Brugha T , Burke J , Cooper JE , Giel R , et al. Schedules for clinical assessment in neuropsychiatry. Arch Gen Psychiatry 1990; 47: 589–93. 2190539 10.1001/archpsyc.1990.01810180089012

[30] Angold A , Cox A , Prendergast M , Rutter M , Simonoff E , Costello EJ , et al. The Young Adult Psychiatric Assessment (YAPA). Duke University Medical Center, 1999.

[31] Goodman R. The Strengths and Difficulties Questionnaire: a research note. J Child Psychol Psychiatry 1997; 38: 581–6. 9255702 10.1111/j.1469-7610.1997.tb01545.x

[32] Gordon D. The concept and measurement of poverty. In Poverty and Social Exclusion in Britain (eds C Pantazis, D Gordon , R Levitas ): 29–69. Policy Press, 2006.

[33] Seaman SR , White IR. Review of inverse probability weighting for dealing with missing data. Stat Methods Med Res 2013; 22: 278–95. 21220355 10.1177/0962280210395740

[34] Hovish K , Weaver T , Islam Z , Paul M , Singh SP. Transition experiences of mental health service users, parents, and professionals in the United Kingdom: a qualitative study. Psychiatr Rehabil J 2012; 35: 251–7. 22246124 10.2975/35.3.2012.251.257

[35] National Institute for Health and Care Excellence (NICE). Depression in Adults: Treatment and Management. NICE, 2022 (https://www.nice.org.uk/guidance/ng222). 35977056

[36] Neufeld SAS , Dunn VJ , Jones PB , Croudace TJ , Goodyer IM. Reduction in adolescent depression after contact with mental health services: a longitudinal cohort study in the UK. Lancet Psychiatry 2017; 4: 120–7. 28087201 10.1016/S2215-0366(17)30002-0 PMC5285445

[37] Singh S , Zaki RA , Farid NDN. A systematic review of depression literacy: knowledge, help-seeking and stigmatising attitudes among adolescents. J Adolesc 2019; 74: 154–72. 31216495 10.1016/j.adolescence.2019.06.004

[38] National Institute for Health and Care Research (NIHR). Public Involvement in Research. NIHR, 2025.

[39] Reupert A , Gladstone B , Helena Hine R , Yates S , McGaw V , Charles G , et al. Stigma in relation to families living with parental mental illness: an integrative review. Int J Ment Health Nurs 2021; 30: 6–26. 33283387 10.1111/inm.12820

[40] Bevan Jones R , Stallard P , Agha SS , Rice S , Werner-Seidler A , Stasiak K , et al. Practitioner review: co-design of digital mental health technologies with children and young people. J Child Psychol Psychiatry Allied Discip 2020; 61: 928–40. 10.1111/jcpp.13258 PMC7611975 32572961

