# Supplementary material for: Formal and informal mental health support in young adults with recurrently depressed parents
Source: BJPsych Open. 2025 Sep 9;11(5):e200. doi: 10.1192/bjo.2025.10819 (PMC12451549; doi:10.1192/bjo.2025.10819)
Supplement: Bevan-Jones et al. supplementary material [file S2056472425108193sup001.docx]

**Supplementary Materials**

**Supplement 1: Measures for support, difficulties, functioning and impairment outcomes in early adult life**

*Mental health support:* Formal support included primary care (general practitioner), secondary care (mental health specialist: psychiatrist, clinical psychologist, mental health nurse), or other formal support (counsellor, social services, student support services, advocate, call line, support worker, wellbeing team, private sleep therapy, hypnotherapist, other therapy). Informal support included self-help (internet-based therapy, self-help group, italk, online meditation, Headspace app, NHS self-help, MoodGYM), internet use (for information or advice), or family member or close friend.

*Psychiatric diagnoses:* For ADHD and MDD, a diagnosis was present if reported by either the parent or the young adult, as had been done in previous waves with this cohort. Parent and child reports were highly correlated^6^.

*Self-harm/suicidal thoughts:* As part of the YAPA during wave 4 interviews, the young adults reported whether they wanted to die, tried to hurt, or kill themselves, thought that life was not worth living, wished they were dead or done anything that made people think that they wanted to die.

*Distress and impairment:* Young adult and parent-reports on the impact supplement of the Strengths and Difficulties Questionnaire (SDQ) were used to assess distress and impairment (at home, school, in friendships or in leisure activities) associated with mental health problems. Five items with responses of “Not at all” (0), “Only a little” (0), “A medium amount” (1) or “A great deal” (2) were summed to give a maximum total score of 10. Those scoring 1 were classed “borderline” and those scoring 2 or more were classed as “abnormal” as recommended previously. Child and parent-reported “borderline” or “abnormal” scores were combined using an either/or approach. Parent and child reports were highly correlated.

*SDQ impairment score:* Responses to the questions on chronicity and burden to others are not included in the impact score. When respondents have answered ‘no’ to the first question on the impact supplement (i.e. when they do not perceive themselves as having any emotional or behavioural difficulties), they are not asked to complete the questions on resultant distress or impairment; the impact score is automatically scored zero in these circumstances.

*Social support:* As part of the interview at wave 4, the young adults were asked to list the people they could most rely on for social support. From this, a binary variable was derived for those with only one or no people to rely on, versus those with two or more people they could rely on.

*Education and employment:* Young adults reported on their education and employment via questionnaire. A binary variable (0=no, 1=yes) capturing whether the young person was not currently in education, employment, or training (NEET status) was derived (NEET=Not currently in full time, part time or occasional work, doing an apprenticeship, in full-time education or self-employed. Includes those who are unemployed, unable to work due to sickness/disability or full/part-time carers). An additional binary variable for whether the young person had not completed a degree and was not currently in university was derived.

**Supplement 2: Missing data and inverse probability weighting (IPW)**

To account for the impact of attrition across the waves (baseline to fourth follow-up), inverse probability weighting (IPW)^28^ was used. This involved weighting the analysis sample by the inverse probability of being missing. Variables measured at baseline (wave 1) were examined as predictors of missingness at wave 4, consistent with previous publications^6^. Variables that predicted missingness at wave 4 were if the family was from a single parent household (B=1.67, p=.041), parent low educational attainment, defined as not achieving GCSE level or equivalent (B=2.39, p=.011), parental low income, defined as a household income of £20,000 or less per annum (B=1.82, p=.002). The presence of psychiatric disorder in the child at baseline (N=1.58, p=.099) was also included in the missingness model as it related to the study outcomes. Minimal missing data on indicators used to derive weights were singly imputed as the modal value (all indicators had <13% missing data). The Hosmer and Lemeshow test indicated that the model was an acceptable fit (X^2^=2.72 (df=4), p=.607). Weights ranged from 1.94 to 6.59.

**Supplement 3: Results from Tables 1-4 in the main text, without IPW**

**Table 1: Prevalence of mental health difficulties and demographic factors**

|  | **Full sample, N (%)**  **or mean (SD)**  N=144 |
| --- | --- |
| **Mental health difficulties** |  |
| Any current psychiatric disorder | 53 (36.8) |
| Any current depressive disorder | 32 (22.2) |
| Any current anxiety disorder | 36 (25.0) |
| Current comorbidity | 22 (15.3) |
| Current self-harm or suicidal thoughts | 17 (11.8) |
| SDQ total difficulties score | 11.5 (5.70) |
| SDQ impairment score | 1.5 (2.21) |
| **Social, educational, and occupational factors** |  |
| Gender (female) | 89 (61.8) |
| Age (years) | 23.4 (2.28) |
| Poor social support | 34 (23.8) |
| Living alone | 17 (12.9) |
| Not in education, employment, or training (NEET) | 19 (14.3) |
| Not completed degree and not currently in university | 56 (39.4) |
| Personal income <£18,000 per annum | 71 (68.3) |

**Footnote for Table 1:** The number with missing data was 15 for the SDQ total difficulties score, 17 for the SDQ impairment score, 1 for social support, 12 for living alone, 11 for NEET, 2 for not completed degree/not currently in university, 17 for parent income, and 40 for personal income.

**Table 2: Support accessed for mental health difficulties in the whole sample, and in those with and without a current psychiatric disorder**

| **Type of support accessed** | **Whole sample**  **N (%)**  N=144 | **Any psychiatric disorder**  **N (%)**  N=53 | **No psychiatric disorder**  **N (%)**  N=91 |
| --- | --- | --- | --- |
| **Formal support** | | | |
| **Medical support**  Primary Care | 30 (20.8) | 21 (39.6)  12 (23.1) | 9 (9.9) |
| Secondary Care | 15 (10.7) |  | 3 (3.4) |
| Medical support total | 37 (25.7) | 26 (49.1) | 11 (12.1) |
| **Other formal support** | 11 (7.6) | 8 (15.1) | 3 (3.3) |
| **Any formal support**  **(total)** | 40 (27.8) | 28 (52.8) | 12 (13.2) |
| **Informal support** | | | |
| **Self-guided support**  Self-help | 7 (4.9) | 6 (11.3)  17 (32.1) | 1 (1.1) |
| Internet | 28 (19.4) |  | 11 (12.1) |
| Self-guided support total | 33 (22.9) | 21 (39.6) | 12 (13.2) |
| **Family member or close friend** | 80 (56.3) | 40 (75.5) | 40 (44.9) |
| **Any informal support (total)** | 83 (57.6) | 41 (77.4) | 42 (46.2) |
| **Any support total**  **(formal or informal)** | 87 (60.4) | 43 (81.1) | 44 (48.4) |

**Footnotes to Table 2:**

The number with missing data was 4 for secondary care and 2 for family member or close friend

Any formal support (total): Primary care, secondary care, or other formal support. Any informal support (total): Self-help, Internet or family member/close friend. Any support total: Any formal or informal support.

**Table 3: Regression analysis on current support accessed by young adults in the whole sample (N=144)**

|  | **Any formal support** | | **Any informal support** | | **Any support** | |
| --- | --- | --- | --- | --- | --- | --- |
|  | **OR**  **(95% CI)** | **P-value** | **OR**  **(95% CI)** | **P-value** | **OR**  **(95% CI)** | **P-value** |
| **Any current psychiatric disorder** | 7.4 (3.27 – 16.61) | <.001 | 4.0 (1.86 – 8.56) | <.001 | 4.6 (2.06 – 10.24) | <.001 |
| **Any current depressive disorder** | 5.3 (2.30 – 12.31) | <.001 | 2.7 (1.10 – 6.40) | .03 | 3.6 (1.36 – 9.33) | .01 |
| **Any current anxiety disorder** | 5.5 (2.43 – 12.45) | <.001 | 4.1 (1.67 – 10.27) | <.002 | 4.5 (1.72 – 11.62) | .002 |
| **Current comorbidity** | 5.1 (1.96 – 13.16) | <.001 | 1.7 (.65 – 4.47) | .28 | 2.5 (.88 – 7.29) | .09 |
| **Current self-harm/suicidal thoughts** | 2.5 (1.15 – 5.24) | .02 | 2.2 (1.06 – 4.65) | .04 | 2.2 (1.02 – 4.61) | .04 |
| **SDQ total difficulties score** | 1.2 (1.07 – 1.25) | <.001 | 1.1 (1.03 – 1.18) | .01 | 1.1 (1.03 – 1.19) | .01 |
| **SDQ impairment score** | 1.6 (1.28 – 1.98) | <.001 | 1.4 (1.10 – 1.72) | .01 | 1.4~~1~~ (1.11 – 1.80) | .01 |
| **Gender (female)** | 2.0 (.92 – 4.48) | .08 | 1.6 (.81 – 3.06) | .18 | 1.7 (.86 – 3.31) | .13 |
| **Age (years)** | 1.0 (0.85 – 1.18) | .96 | 1.0 (0.86 – 1.15) | .94 | 1.0 (0.88 – 1.19) | .74 |
| **Poor social support** | 1.7 (.66 – 4.23) | .28 | 2.1 (.96 – 4.58) | .06 | 1.8 (.82 – 3.91) | .14 |
| **Living alone** | 2.0 (.69 – 5.69) | .20 | .99 (.35 – 2.78) | .98 | 1.6 (.53 – 4.85) | .41 |
| **Not in education, employment, or training (NEET)** | 2.8 (1.02 – 7.49) | .05 | 1.6 (.56 – 4.44) | .39 | 1.9 (.64 – 5.62) | .25 |
| **Not completed degree and not currently in university** | 0.4 (.21 - .93) | .03 | 0.7 (.35 – 1.37) | .29 | 0.6 (.28 – 1.15) | .12 |
| **Personal income <£18,000 per annum** | 1.4 (.51 – 3.64) | .54 | 1.5 (.63 – 3.32) | .39 | 1.6 (.71 – 3.76) | .25 |

**Table 4**: **Regression analysis on current support accessed by young adults with a psychiatric disorder (N=53)**

|  |  | **Any formal support** | | **Any informal support** | | **Any support** | |
| --- | --- | --- | --- | --- | --- | --- | --- |
|  | **N (%) or mean (SD)** | **OR**  **(95% CI)** | **P-value** | **OR**  **(95% CI)** | **P-value** | **OR**  **(95% CI)** | **P-value** |
| **Current comorbidity** | 22 (41.5) | 1.5 (.51 – 4.5) | .44 | 0.4 (.11 – 1.53) | .19 | 0.7 (.16 – 2.61) | .55 |
| **Current self-harm/suicidal thoughts** | 30 (56.6) | 0.8 (.29 – 2.29) | .64 | 1.4 (.39 – 5.13) | .60 | 0.8 (.21 – 3.32) | .81 |
| **SDQ total difficulties score** | 15.1 (5.53) | 1.1 (1.00 – 1.28) | .05 | 1.0 (.90 – 1.15) | .82 | 1.0 (.91 – 1.19) | .53 |
| **SDQ impairment score** | 2.9 (2.80) | 1.5 (1.10 – 1.96) | .01 | 1.2 (.90 – 1.64) | .20 | 1.2 (.89 – 1.66) | .22 |
| **Gender (female)** | 33 (62.3) | 1.2 (.39-3.65) | .75 | 1.9 (.52 – 7.10) | .32 | 1.9 (.47 – 7.49) | .38 |
| **Age (years)** | 23.1 (2.26) | 1.0 (0.80 – 1.30) | .86 | 0.9 (0.69 – 1.23) | .57 | 1.0 (0.70 – 1.29) | .95 |
| **Poor social support** | 11 (20.8) | 2.3 (.59 – 9.2) | .23 | 2.4 (.57 – 10.35) | .23 | 1.9 (.40 – 8.88) | .43 |
| **Living alone** | 8 (16.3) | 1.8 (.37 – 8.30) | .48 | 0.8 (.15 – 4.92) | .85 | 2.0 (.21 – 18.16) | .55 |
| **Not in education, employment, or training (NEET)** | 13 (26.0) | 1.2 (.35 – 4.37) | .75 | 0.3 (.08 – 1.28) | .11 | .44 (.10 – 1.89) | .27 |
| **Not completed degree and not currently in university** | 24 (46.2) | 2.2 (.73 – 6.78) | .16 | 2.0 (.52 – 7.72) | .32 | 4.4 (.83 – 23.23) | .08 |
| **Personal income <£18,000 per annum** | 25 (73.5) | 0.2 (.03 – 1.11) | .07 | 1.5 (.22 – 10.04) | .68 | 1.5 (.22 – 10.04) | .68 |

**Supplement 4:**

**Table: Sensitivity analysis - regression analysis on current informal support accessed by young adults in the whole sample and in those with disorder - both when excluding family and friends support** (with IPW)

|  | **Any informal support in the whole sample**  (excluding friends and family) | | **Any informal support** **in those with disorder**  (excluding friends and family) | |
| --- | --- | --- | --- | --- |
|  | **OR**  **(95% CI)** | **P-value** | **OR**  **(95% CI)** | **P-value** |
| **Any current psychiatric disorder** | 4.3 (2.48-7.35) | <.001 | - | - |
| **Any current depressive disorder** | 2.7 (1.58-4.74) | <.001 | - | - |
| **Any current anxiety disorder** | 5.1 (2.92-8.83) | <.001 | - | - |
| **Current comorbidity** | 2.8 (1.50-5.04) | .001 | 1.1 (0.53-2.21) | .82 |
| **Current self-harm/suicidal thoughts** | 0.6 (0.26-1.49) | .29 | 0.29 (0.11-0.74) | .01 |
| **SDQ total difficulties score** | 1.1 (1.08-1.18) | <.001 | 1.1 (1.00-1.15) | .04 |
| **SDQ impairment score** | 1.3 (1.13-1.42) | <.001 | 1.1 (0.98-1.29) | .11 |
| **Gender (female)** | 0.8 (0.48-1.34) | .40 | 0.8 (0.40-1.69) | .82 |
| **Age (years)** | 0.9 (0.81-1.02) | .10 | 0.8 (0.67-0.94) | .01 |
| **Poor social support** | 1.9 (0.97-3.68) | .06 | 2.5 (1.02-6.15) | .05 |
| **Living alone** | 0.8 (0.36-1.80) | .60 | 0.3 (0.09-0.89) | .03 |
| **Not in education, employment, or training (NEET)** | 2.0 (1.03-3.75) | .04 | 0.8 (0.34-1.66) | .48 |
| **Not completed degree and not currently in university** | 0.7 (0.40-1.13) | .13 | 1.3 (0.62-2.59) | .51 |
| **Personal income <£18,000 per annum** | 2.9 (1.33-6.45) | .01 | 2.4 (0.85-6.81) | .10 |

**Supplement 5: Table: Qualitative responses on satisfaction with help received from services**

**Question: If you have ever used services for help with mental health, were you satisfied with the help you received? (Yes/No) Why?**

| **Positive or favourable** |
| --- |
| *Understanding, listening:*  Taken seriously, understanding  GP v helpful + understanding of MH, quick referral v good + effective counsellor  Just good, listened  Helped rationalise it + get to bottom of feeling. Private so happy with speed. |
| *Independent person:*  Being able to talk to someone impartial about it.  It worked, needed to speak to someone not in the family and now feeling normal. |
| *Improvement:*  Relief at the time  It's worked, happy with speed + ability to get an appointment.  Very good counselling  Makes you feel hopeful  Helped feel better.  Helped set up mentor, changed life in last year |
| *Medication:*  Worries for her, medication has helped & not needed anything else |
| **Negative** |
| *Waiting times:*  Long waiting times  Side effects from medication. Long waiting lists everywhere.  Put on 2 year waiting list to talk to people  Could have done with more help, one month delay before got help |
| *Relationship with professionals:*  NHS just isn't able to deal with problems like that [MH problems]. Bad relationship with psychiatrist had to push for regular sessions and the right therapy. felt let down.  College counsellor - no hadn't heard of my OCD type, somewhat satisfied with help received but tended to deal with it on own.  Hated it, didn't like speaking to people. CAMHS - felt people were against me and made me feel small possibly because young.  All terrible, sleep therapist wanted to have sex with mother as payment. Didn't grasp what I was talking about.  Somewhat - not very good at talking |
| *Not enough or poor support:*  CAMHS - not helpful, not coping strategies, didn't get medication until had to confess hear voices. Crisis team - had breakdown and cut wrists, went A&E and just asked if okay and sent away. CPN - not enough support, need additional.  Been very disjointed.  Tried reading book, gained nothing, felt pointless seeing doctors.  Always spoke to counsellor or doctor, felt needed a psychologist.  Self-help group - wasn't helpful, too much Facebook.  Therapy at 16 not great.  They should be doing more counselling. |
| *Lack of follow-up:*  Psychiatrist said they were going to follow up, but received no follow-up regarding CBT. If chased, would have gone to CBT but not given an option so went to GP to go on medication. Went to GP to go through with fluoxetine as no CBT option.  No FU, feeling of concern. Looking for people more vulnerable/ at risk, I was not actively self harming etc so not a priority. Felt held off for a few months even though felt like for a year. Told to wait 3 months and come back. |
| *Medication:*  Just wanted to medicate me.  Offered medication as first option – insensitive.  Feels rushed out of door at GP, medication first rather than talking through things.  Some of it, don't think medication works it just masks the problem |
| **Mixed / Neutral** |
| *Unhappy with primary care, better support from others:*  GP not v helpful - when decided to come off antidep no follow up, put on repeat prescription + not contacted since. Student support great.  GP didn't listen/care. Counsellor good, helped explain self-healing.  Counsellor was really good. GP not helpful. Family therapy - helpful.  GP - no. Counselling - yes, but too late by the time he saw them, didn't really need it then.  Got all support needed, counsellor v understanding, regular sessions. GP always a problem to get an appointment. Confusion this year about medical letter, bad communication. |
| *Unhappy with CAMHS, better support from others:*  CAMHS didn't help, saying things like he already knew mood gym -no, made worse, asked questions and the answers he wanted weren't options; others, yes – satisfied.  Alright, CAMHS - not great.  Educational psychologist in CAMHS not helpful, happy with more recent help. Not satisfied with counsellor, last session waiting for 2 hours. GP – happy. |
| *Inconsistent support, but better recently:*  Only recently felt supported. Was with CAMHS from age 8-17 then the support stopped abruptly. Go from seeing someone for 10 years to then nothing.  Didn't feel got help often enough. Not taken seriously when younger. Happy with GP now.  Sometimes, not hugely. Now v good. Counselling okay. Often not dealt with problems. |
| *Other mixed experiences:*  Counselling no - group therapy been helpful  CBT was good, online therapy wasn't good  Sort of satisfied. GP referred to consultant regarding contraception and emotions.  All fine, one counsellor didn't use age-appropriate methods e.g. used dolls.  Have researched it but never went through with it (online therapy) |

**Supplement 6 - Focus group with young adults on access to support**

A focus group session was held with young adults from the EPAD study and a member of the National Centre for Mental Health youth advisory group via videoconferencing to explore access to various types of support, enrich the study findings and help guide further research and practice. The group was facilitated by RBJ, EM and a research assistant, following a pre-prepared topic guide, which included the following questions.

- Opening discussion point – any general comments about young adult’s access to support or help-seeking for mental health (MH) difficulties?
- Do you think young adults of your age go for help if they have MH difficulties?
- Where would you/they go for help? (e.g. family/peers, GP, charities, internet…)
- A common finding is that lots of young adults who are experiencing MH difficulties, do not get or seek help for their MH. Why do you think that might be? During pandemic and pre-pandemic?
- What barriers are there to getting/seeking help for MH? How could we overcome these?
- What facilitators to seeking help for MH are there?
- How do you feel about getting help from outside MH services, for example through charities? Compared to MH services? Advantages? Disadvantages?
- How do you feel about using internet or mobile phone resources for MH difficulties? Compared to face-to-face? Advantages? Disadvantages?
- Do you think that having someone in the family (or someone close to you) with MH difficulties affects whether someone might go for help for their own MH?
- Do you think that where/how people go for help change as they get older e.g. from teenage years to young adulthood?

The session was held in 2021 and lasted approximately 90 minutes. Mentimeter was used to gather answers to specific questions and to encourage discussion. We aimed for a balance in terms of the gender and age of participants. The group was digitally audio-recorded and transcribed; participants could also contribute through the videoconferencing platform’s ‘chat function’ or by emailing researchers separately.

The transcript was analysed using a thematic analysis approach. This is a process of identifying, analysing, reporting, and interpreting patterns or themes. To ensure the reliability of coding, the transcript was coded by RBJ and double-coded independently by EM. Initial ideas on the coding framework were discussed among the team. Transcripts were examined to identify the key themes and associated subthemes.

Six people agreed to participate in the focus group, however, two did not attend. Of the four participants, two identified as female, one as male, and one as non-binary. One was aged 21-23 and three were aged 24-27. Three were working full-time and one was in full-time education. Three had experienced mental health difficulties, and all had sought support/advice (e.g. health services, helplines, websites) for such difficulties. The themes, subthemes and verbatim examples are presented in the table below.

To address barriers to accessing support and help-seeking, participants suggested increasing the number of role models for young adults, providing clear guidance on support pathways, promoting a ‘positive narrative’ around mental health, and developing educational and self-help resources (e.g. through charities and health services). Further involvement work and mixed-methods research is required to build on these findings.

**Table: themes, subthemes, and quotes from focus group - on access to support for mental health difficulties**

| **Themes & subthemes** | **Verbatim examples** |
| --- | --- |
| **Key theme 1: Young adults access a range of online and informal sources of support** |  |
| Internet:   - often first port of call but will not suit everyone - a key advantage is the ability to access more specialised help - more likely to use than when younger | *“I think* *it’s very personal. It can vary massively between people. Some people will be much happier online, some people find it much harder, so I* *don’t think there’s a blanket answer for everyone.”*  *“Finding help for more specific conditions or specific circumstances so they’ll feel people understand them more.”*  *“As a young adult I’d be more likely to use the Internet, approach things that way, that context wasn’t available when I was a teenager, so I don’t know if that’s an age change or just a world change.”* |
| More virtual/remote sessions | *“[I think how people receive support has fundamentally changed]; anyone who had been receiving counselling over the past year would have to have done it virtually. In many ways for some people,* *that’s going to be great because I know it can often be harder to open up in person...but it may also be more difficult for someone else.”* |
| Apps such as Headspace, mindfulness, yoga. Mind website. |  |
| Local youth mental health charity | *“[The charity* *are really good at outreach and stuff and I know a lot of people will end up in it because their friends’ friends mentioned it or something.]* *Because* *it’s not medicalized, people feel happier to access it.”* |
| Someone they know/trust – parent, guardian, or friend (or someone at school) – often first port of call |  |
| Paradox during pandemic regarding public health measures to protect against the virus versus mental health advice | *“There’s a weird paradox with things that have happened over the course of the last year [during the COVID-19 pandemic] in that more common mental conditions like anxiety and depression, the things that you’re encouraged to do like get out and about, socialise, physical contact, exercise, that’s all been inverted.”* |
| **Key theme 2: Facilitators for help-seeking for mental health difficulties** |  |
| There are more role models | *“I think broadly yes [young people would seek help for their mental health difficulties] but I think* *that’s down to a lot of people who are famous or are in the public eye, who are making documentaries or speaking about it more. They’re breaking down the stigma around it.”*  *“I think that getting publicity around the fact that it’s ok to speak to people, it’s ok to feel certain ways...and advertising the fact that you can speak to people, and you can get help.”* |
| More talking about mental health in general | *“There’s...been a change in the conversation, society-wise...”* |
| There has been more education of doctors/practitioners regarding mental health in general and better quality of care | *“...I think* *it’s also the general quality of care. The education of doctors, which sounds ridiculous, but as the first points of contact, it seems that there this a lot more understanding and a lot more clear signposting.”* |
| More understanding of psychological approaches and less reliance on medication | *“There’s more understanding of what needs to be done, rather than in the past (and in my personal experience and other people I know), it’s been medicate first and counsel later. It works, am sure, for some people but without counselling alongside ‘take anti-depressants’, it’s quite pointless.”* |
| **Key theme 3: Barriers to help-seeking: difficulties in articulating & understanding experiences & difficulties** |  |
| Difficult to convey how and why they feel as they do | *“It’s hard to tell someone when you don’t actually understand yourself.”*  *“When I was a teenager, I didn’t have the facilities to do that [speak about it].”* |
| Can take time to acknowledge own difficulties | *“I’ve seen it a lot with guys at university in the sense of ‘it’ll pass’ or self-medicate with alcohol, drugs, whatever – finding any reason to not do it. That requires a degree of self-acknowledgement but that’s a really hard thing to deal with.”*  *“There are still prevailing ideas of who has mental illnesses… ‘I don’t have a problem because the people that have problems are X, Y, Z not me’.”* |
| Underplaying/minimising seriousness of situation | *“(hard to)…tell someone in such a way that impresses the seriousness on that person. And to make sure that person – the doctor or whoever – takes it seriously too.”* |
| Scope to become more articulate about feelings as they get older | *“I reached out to my GP for help when I was younger and as I’ve got older, I’ve outsourced a therapist …. When you’re younger you don’t really realise that because you just think you’re getting help but as you get older you think, well maybe this isn’t the right person for me or maybe I should be seeking help somewhere else or with someone different. So, I think as you get older, you learn those things, but you wouldn’t have known that if you’d just gone to your GP.”* |
| **Key theme 4: Barriers to help-seeking: societal & service/support factors** |  |
| Lack of education & awareness of sources of help | *“Probably comes back to education again, they don’t know where to get the right help for them, or where to start.”*  *“I do feel that some people just don’t know that the services are there to use.”* |
| Stigma | *“[Around the society aspect as well and] the stigma around mental health – that you shouldn’t talk about it, and you don’t have mental health issues. All that is drilled into you. And if you do, then you’re not cool or anything like that.”* |
| Giving up (pressure on services / waiting lists) | *“I think a feeling of ‘what’s the point?’. It’s no secret how long waiting lists are, a lot of young people just can’t be bothered.”*  *“There’s a lot to be said for narrative, I think. I remember when I was at Uni, there was this whole narrative that waiting times for counselling were just far too long. During my time there, it got so much better, but the narrative remained the same.”* |
| Worries about bias/prejudice in the system | *“LGBTQ+ people, or trans people won’t seek help because they’re worried about the transphobia, they’re going to experience in the healthcare system. Or what will be conflated with them and their mental health problems.”* |
| **Key theme 5: Effect of seeing someone close to you experience difficulties** |  |
| Very complicated and with lots of variables – no easy answer | *“There’s a lot of variables with something like this. If it’s a parent, it depends on the relationship with the parent, it depends on the person, it depends on the parent as well.”* |
| Depends on how the person managed it | *“I think it would* ***depend on how that person dealt with their mental health****. It comes back to role models again; if they sought help and managed it well then it might encourage that young person to do the same but if they didn’t and they struggled really badly with it then, they might not know all the options. I think it all depends on how they’ve managed it.”* |
| Effect of other people’s negative experiences | *“They’ve seen friends or colleagues try to get help and fail or try to get help and it hasn’t helped. They might be discouraged from trying to find the same kind of help.”* |
